# The Inclusion of *Hermetia Illucens* Larvae Meal in the Diet of Laying Hens (Hy‐Line Brown) Affects the Caecal Bacterial Composition and Diversity

**DOI:** 10.1002/vms3.70650

**Published:** 2025-10-21

**Authors:** Tiziana Maria Mahayri, Elie Atallah, Jakub Mrázek, Fulvia Bovera, Giovanni Piccolo, Giuseppe Moniello, Kateřina Olša Fliegerová

**Affiliations:** ^1^ Laboratory of Anaerobic Microbiology Institute of Animal Physiology and Genetics Czech Academy of Science Prague Czech Republic; ^2^ Department of Veterinary Medicine University of Sassari Sassari Italy; ^3^ Department of Veterinary Medicine and Animal Production University of Napoli Federico II Napoli Italy; ^4^ Department of Veterinary Medicine and Animal Sciences University of Milan Via dell'Università Lodi Italy

**Keywords:** black soldier fly, insect meal, intestinal microbiota, laying hens

## Abstract

**Background:**

The poultry industry is increasingly looking for sustainable feed ingredients which support animal productivity. Insects, such as the larvae of *Hermetia illucens*, represent a promising alternative protein source that has a positive effect on gut health.

**Aim:**

The aim of this study is to evaluate the effects of a partial replacement of soybean meal with *H. illucens* larvae meal on the caecal microbiota of laying hens.

**Methodology:**

A total of 162 hens were divided equally into three treatment groups: a control group (C) with a diet containing corn–soybean meal and two treatment groups (HI25, HI50) in which 25% and 50% of the soybean meal protein was replaced by *H. illucens* larvae meal protein. At 40 weeks of age, 30 animals (10 per group) were slaughtered and the bacterial community of the caecal content was analysed by high‐throughput sequencing using the V4–V5 region of the 16S rRNA gene. The DNA was extracted using the PowerSoil DNA Kit, the library preparation was performed using the NEBNext Fast DNA Library Prep Set kit and sequencing was performed using the Ion Torrent PGM. The bacterial diversity was assessed by alpha and beta diversity indices, and the differential abundance of taxa was determined using LEfSe analysis.

**Results:**

Firmicutes and Bacteroidetes were the dominant phylum in all groups. Alpha diversity indices showed no significant differences between diets, however, beta diversity measures showed statistical dissimilarities between the three studied groups. Several beneficial genera, including *Alistipes*, *Christensenellaceae* R‐7 group, *Parabacteroides*, *Butyricimonas* and *Parasutterella*, were enriched in the HI50 group, while *Lactobacillus*, *Bifidobacterium*, *Blautia* and the opportunistic pathogen *Enterococcus* were reduced.

**Conclusion:**

The consumption of *H. illucens* larvae meal showed beneficial effect on the microbiota of the caecum of laying hens, with 50% replacement showing the strongest positive effects, suggesting that this is the most effective amount under the given conditions. Further research should explore microbial functions and long‐term impacts, and validate the optimal levels of insect meal inclusion.

## Graphical abstract

This study evaluated partial replacement of soybean meal with *Hermetia illucens* larvae meal in laying hens’ diets. While alpha diversity was unaffected, beta diversity revealed distinct microbial profiles among treatments. A 50% replacement enriched beneficial genera (*Alistipes*, *Parabacteroides*, *Butyricimonas*) and reduced potentially harmful taxa (*Enterococcus*), indicating positive modulation of caecal microbiota.

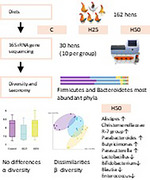

## 1 Introduction

The production of food from animal sources has risen up greatly in recent years, and poultry farming plays an important and irreplaceable role in this area. Based on FAO data, world egg production has expanded by 150% in the last three decades (FAO 2022). This fact is also related to the increased demands on laying hens (El‐Sabrout et al. 2022). Nutritional modifications are one way to influence the production and health status of these birds. Diet is known to have a significant impact on poultry (Alagawany et al. 2021; Zampiga et al. 2021) and intestinal microbiome, which plays a crucial role in feed digestion and is therefore intensively studied (Aruwa and Sabiu 2024; Kogut 2022). The gut microbiome of poultry is a complex ecosystem, with the caecum being the most densely populated (Józefiak et al. 2020; Pan and Yu 2014; S. Wei et al. 2013). Bacteria in the caecum of laying hens have a significant impact on nutrient utilisation (Dai et al. 2020), production performance (Wang et al. 2020) and egg quality (Zhan et al. 2019). They are involved in the regulation of immunological processes and defence against infections caused by potential pathogens such as *Salmonella*, *Escherichia* or *Campylobacter* (Józefiak et al. 2020).

Research has mainly focused on the composition of the intestinal microbiota of broilers (Bjerrum et al. 2006; Gong et al. 2002; Rinttilä and Apajalahti 2013; Shang et al. 2018; Stamilla et al. 2021). However, laying hens have different genotypes and nutrient requirements than broilers, which can affect the composition of the microbiota. The composition of the caecal microbiota also changes with age, but diet appears to have the greatest influence on its structure (Kogut 2022; Mahayri et al. 2025).

Recently, the use of insects as an alternative source of protein has been increasingly applied in poultry farming (Józefiak et al. 2020; Liceaga et al. 2022). *Hermetia illucens* larvae, also known as black soldier fly (BSF), have widespread use on a global scale, due to their high content of protein and fats, beneficial profile of essential amino acids, vitamins and adequate amount of minerals (El‐Hack et al. 2020; Barragan‐Fonseca et al. 2017; Zulkifli et al. 2022). Consequently, a good nutritional value of BSF larvae meal has a positive influence on feed conversion, growth performance and quality of end products (Slimen et al. 2023). The high content of chitin and lauric acid is associated with natural antibiotic properties, reducing harmful bacteria (Lee et al. 2018; Xia et al. 2021). Moreover, BSF larvae production is environmentally friendly and economically beneficial due to the capability to convert substantial amounts of waste organic matter into fertilizer (Ferronato et al. 2024) or edible proteins and fats (Hayat et al. 2024; Raksasat et al. 2020; Siddiqui et al. 2022). All these factors indicate the possibility and advantage of using BSF larvae meal in poultry nutrition (El‐Hack et al. 2020; Attia et al. 2023; Barragan‐Fonseca et al. 2017; Zulkifli et al. 2022).

Research in this area is mainly focused on broilers, and most studies reported positive effects of BSF meal on feed intake (Józefiak et al. 2018), body weight gain (Moula et al. 2018; Dahiru et al. 2016), immune activities (de Souza Vilela et al. 2021; Lee et al. 2018) and caecal bacterial composition (Biasato et al. 2020; Józefiak et al. 2018). However, the results depend on the amount of insect meal inclusion (He et al. 2021), and some studies showed no significant impact (Cullere et al. 2016).

In contrast, less information is available on the influence of BSF larvae meal on laying hens. Some studies reported beneficial effects, such as increased eggshell thickness and higher caecal microbiota diversity (El‐Hack et al. 2020; Borrelli et al. 2017; Kawasaki et al. 2019; Yan et al. 2023). On the other hand, no effect of supplementation with BSF larval meal on egg weight (Bovera et al. 2018; Mwaniki et al. 2018), feed intake (Kawasaki et al. 2019) and feed conversion ratio (Bovera et al. 2018) was demonstrated. Ruhnke et al. (2018) even described significantly lower egg weight and shell thickness of eggs from BSF larvae fed hens. These contradictory results indicate that the effects of BSF larvae meal on laying hens, and especially their caecal microbiome, are still poorly understood. Given these inconsistencies, there is a clear need for more detailed studies to evaluate the impact of BSF larvae meal on the caecal bacteriome of laying hens.

Therefore, the present study aimed to investigate the effects of partial replacement of soybean protein (25% and 50%) with *H. illucens* larvae meal on the caecal bacteriome of Hy‐line Brown laying hens.

## 2 Materials and Methods

### 2.1 Ethical Statement

The hens were treated humanely in accordance with the European Union (EU) legislation Directive 2010/63/EU on the protection of animals used for experimental and other scientific purposes. The experimental procedures were approved by the Institutional Animal Care and Use Committee of the Department of Veterinary Medicine and Animal Production of the University of Napoli Federico II, Italy (prot. no. 2017/0017676). The trial was carried out in a private laying hen farm located in Sardinia (Italy), and the study design, experimental procedures and animal handling were performed in accordance with the ARRIVE (Animal Research: Reporting of In Vivo Experiments) guidelines (du Sert et al. 2020).

### 2.2 Animals and Diet

A total of one hundred sixty two 16‐week‐old Hy‐line Brown hens with an average weight of 1.41 ± 0.13 kg were housed for 20 weeks in modified cages (800 cm^2^/hen) under controlled conditions of temperature and humidity. The birds were exposed to natural lighting (approximately 15:9 h light:dark) and received feed and fresh water ad libitum. The hens were divided evenly and randomly into three experimental groups (nine replicates, six hens per replicate) and were fed three isoproteic and isoenergetic diets developed to fulfill the hens' requirements according to the Hy‐line Brown commercial line management guide (Guide 2016). The control group (C) was offered a corn–soybean meal (SBM) based diet and in the experimental groups HI25 and HI50, 25% and 50% of the protein content of the soybean meal was replaced by the partially defatted *H. illucens* (HI) larvae meal protein purchased from a European company specialized in insects as a nutritional source (HI, Hermetia Deutschland GmbH & Co. KG, Amtsgericht Potsdam, Germany). The replacement levels (25% and 50%) were selected based on previous studies on poultry reporting positive effects of partial substitution without impairing performance (Loponte et al. 2017; Secci et al. 2018). The samples of the protein sources (SBM and HI) and diets were analysed as described in Bovera et al. (2018). Proximate composition, mineral and essential amino acid composition (% as fed) of the insect and soybean meals are shown in Table S1. The ingredients and chemical‐nutritional characteristics of diets are given in Table S2.

### 2.3 Samples Collection

At 40 weeks of age, 10 hens from each treatment group were randomly selected and slaughtered in a specialised slaughterhouse. After evisceration of the entire gastrointestinal tract, the lumen contents of the sterilely separated caeca were collected in sterilised micro‐centrifuge tubes (2.0 mL, Eppendorf), transported to the laboratory on ice and frozen at −80°C. The samples were lyophilised using the Heto PoweDdry LL3000 freeze dryer (Thermo Fisher Scientific, Wilmington, DE, USA), and transported to the Institute of Animal Physiology and Genetics of the Czech Academy of Sciences (Prague, Czech Republic) for further analysis. The characteristics of the animals and the weight of the samples for DNA extraction are listed in Table S3. The caeca with small amounts of intestinal content were excluded, and nine samples per each group were further used for the microbiota analysis.

### 2.4 DNA Extraction and PCR Amplification

The genomic DNA was extracted from the dry caecal samples using PowerSoil DNA Kit (QIAGEN, Hilden, Germany), according to the manufacturer's protocol. The concentration and quality of the DNAs were measured using a NanoDrop 2000c UV–Vis spectrophotometer (Thermo Scientific, Wilmington, DE, USA) and the DNA was stored at −20°C until further use. The V4–V5 region of the bacterial 16S rRNA gene was amplified with the primer pair BactBF and BactBR (Fliegerova et al. 2014) using the EliZyme HS FAST MIX Red Master Mix (Elisabeth Pharmacon, Brno, Czech Republic). The thermal cycling conditions included a denaturation step for 5 min at 95°C, followed by 25 cycles of 30 s at 95°C, 30 s at 57°C and 30 s at 72°C, a final elongation step at 72°C for 5 min. The length and quality of the amplicons were checked by 1.5% agarose gel electrophoresis. The Monarch PCR and DNA Cleanup Kit (New England BioLabs, Ipswich, MA, USA) was used for amplicon purification, and their concentration was measured using a NanoDrop 2000c UV–Vis spectrophotometer (Thermo Scientific, Wilmington, DE, USA).

### 2.5 Library Preparation and Next‐Generation Sequencing

Library preparation was performed using the NEBNext Fast DNA Library Prep Set kit (New England BioLabs, Ipswich, MA, USA) and the Ion Xpress Barcode Adapters 1–96 kit (Thermo Fisher Scientific, Waltham, MA, USA) as previously described (Atallah et al. 2023). Libraries were quantified using the KAPA Library Quantification Kit (KAPA Biosystems) on a QuantStudio 3 Real‐Time PCR System (Thermo Fisher Scientific, Waltham, MA, USA), and subsequently pooled in equimolar ratios. Template amplification was carried out in the Ion OneTouch 2 instrument using emulsion PCR with the Ion PGM HiQ View OT2 400 kit (Thermo Fisher Scientific, Waltham, MA, USA). The enriched template was sequenced with the Personal Genome Machine (PGM) System (Thermo Fisher Scientific, Waltham, MA, USA) using the Ion PGM Hi‐Q View Sequencing Solutions kit and the Ion 316 Chip v2 BC according to the manufacturer's instructions.

### 2.6 Bioinformatic and Statistical Analysis

The sequence data retrieved from the Ion Torrent software in FASTQ format were analysed using Qiime2 version 2020.2 software (Bolyen et al. 2019). The sequences were trimmed, quality filtered and denoised using DADA2, and chimeras were removed (Callahan et al. 2016). The dataset was subsampled to a minimum of 6000 reads per sample to obtain an equal sampling depth. The rarefaction curves reached a plateau, allowing cross‐sample comparison (Figure S1). The sequences were clustered into Amplicon Sequence Variants (ASVs) by VSEARCH, and taxonomic assignment was performed using a BLAST search against the SILVA database (version 138) with a 97% threshold (Rognes et al. 2016). The diversity of bacterial communities was assessed by alpha and beta diversity metrics. Alpha diversity was evaluated using Faith's phylogenetic diversity, Pielou's evenness index and Shannon entropy. The groups were compared using the Kruskal–Wallis *H* test. Beta diversity was assessed using Jaccard's distance matrix. Principal coordinate analysis (PCoA) was used for visualisation, and results were presented using EMPeror plot (Vázquez‐Baeza et al. 2013). The permutational multivariate analysis of variance (PERMANOVA) with 999 permutations was performed to determine the statistical differences between the groups. The PERMDISP test was carried out to evaluate the homogeneity of dispersions between bird groups. Linear discriminant analysis (LDA) with an effect size (LEfSe) algorithm (Segata et al. 2011) was performed using the Galaxy web module (http://huttenhower.sph.harvard.edu/galaxy/) to determine the significantly differentially abundant taxa. The following parameters were used: alpha = 0.05 and a minimum LDA score = 2.0. The sequence information was deposited in the Sequence Read Archive under accession number: PRJNA962558.

## 3 Results

### 3.1 Effect of BSF Larvae Meal on Caecal Bacterial Diversity

**FIGURE 1:**
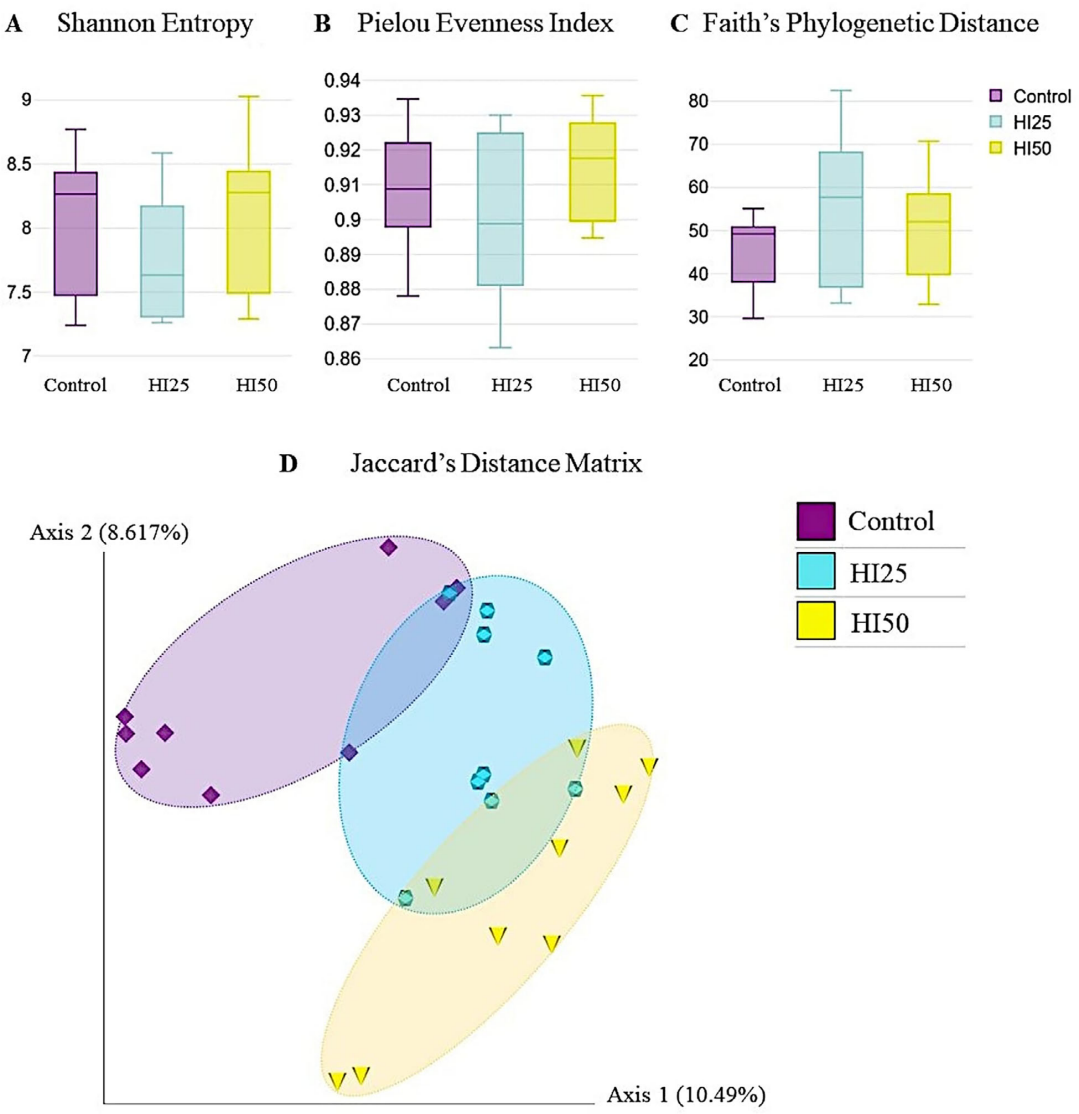
Comparison of alpha and beta diversity of bacterial communities in the caecum of three groups of hens fed different diets (Control, HI25 and HI50). The diversity indices show (A) Shannon Entropy, (B) Pielou Evenness Index and (C) Faith's Phylogenetic Distance with no significant differences among the groups. (D) Principal Coordinate Analysis (PCoA) shows the Jaccard's distance matrix among the caecal bacterial community compositions of hens fed different diets. Each dot represents one sample. The percentage of variation explained by the plotted principal coordinates is indicated on the axes.

The influence of the inclusion of BSF larvae meal in the hens' diet on the bacterial community in the caecal samples was analysed qualitatively and quantitatively for species richness, evenness and phylogenetic diversity. The alpha diversity indices, including Shannon entropy, Pielou's evenness index and Faith's phylogenetic distance, showed no significant differences among the three groups of birds (*p* > 0.05, Figure 1A–C). The beta diversity, evaluating the similarities/dissimilarities of bacterial communities of hens fed different diets, was assessed using Jaccard's non‐phylogenetic distance matrix and showed a significant impact of insect meal on caecal bacteria. The spatial separation of the samples is represented in the PCoA plot (Figure 1D). Statistical analysis revealed significant differences among the three groups of hens (PERMANOVA *p* < 0.05). However, the results may be partially influenced by the high variability among the groups, which was assessed by the PERMDISP test of homogeneity of multivariate dispersions (Table 1).

**TABLE 1:** Permutational multivariate analysis of variance (PERMANOVA) and dispersion (PERMDISP) based on the Jaccard distance matrix.

	Jaccard distance matrix	
Diet	PERMANOVA *p* value	PERMDISP *p* value
Control vs. HI25	0.001^a^	0.001^a^
Control vs. HI50	0.001^a^	0.231
HI25 vs. HI50	0.003^a^	0.013^a^

^a^ Significant difference (*p* < 0.05).

### 3.2 Effect of BSF Larvae Meal on the Caecal Bacterial Composition

**FIGURE 2:**
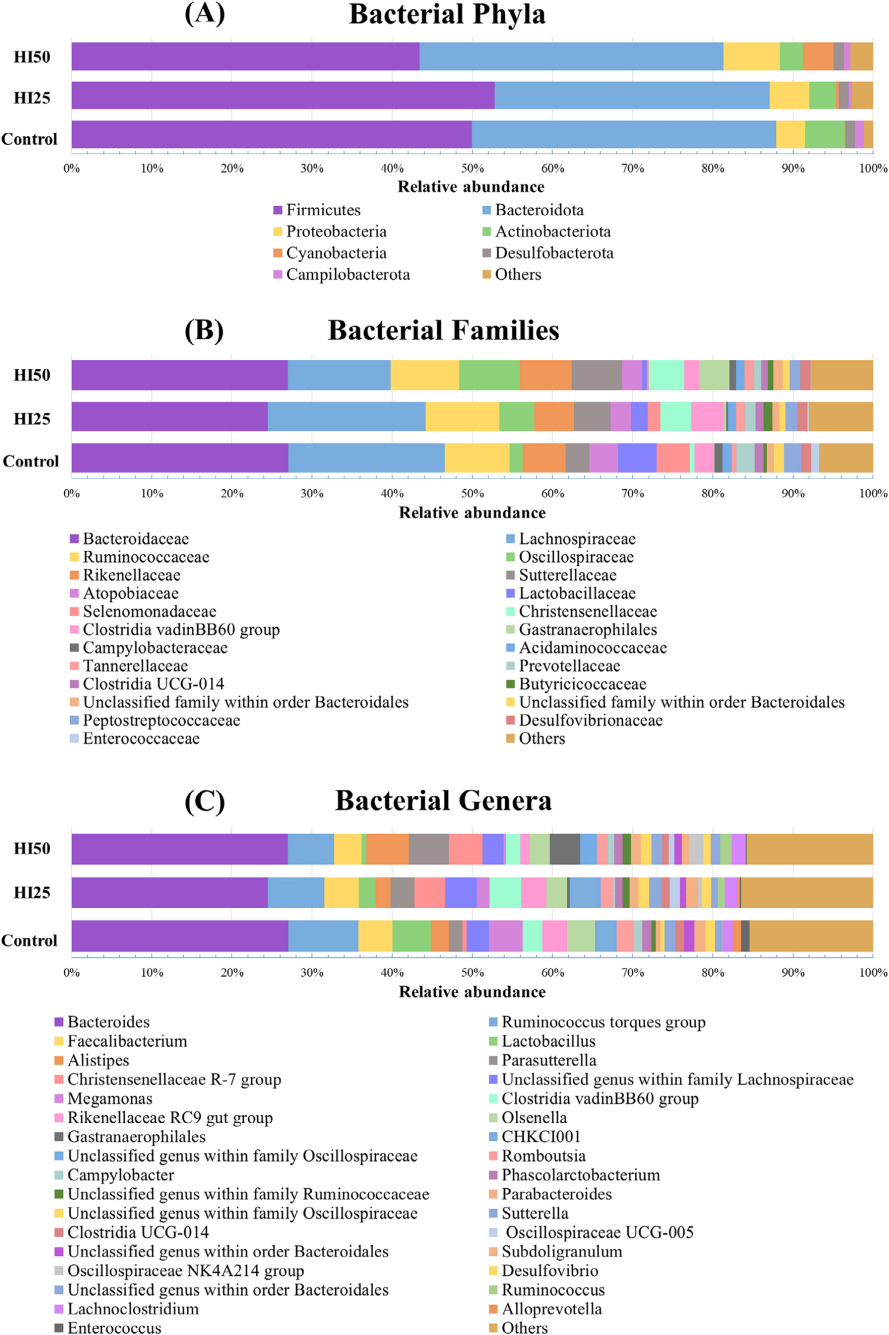
Relative abundance of caecal bacteria in three groups of hens fed different diets (Control, HI25 and HI50) illustrated at phylum (A), family (B) and genus (C) level. Taxa with a relative abundance lower than 1% are grouped as ‘Others’.

A total of 14 phyla, including 249 bacterial phylotypes, were detected in the entire dataset, but only 7 of them, including Firmicutes, Bacteroidetes, Proteobacteria, Cyanobacteria, Actinobacteria, Campilobacterota and Desulfobacterota had a meaningful relative abundance (> 1%). The abundances of phyla Deferribacterota, Verrucomicrobiota, Spirochaetota, Patescibacteria, Synergistota, Elusimicrobiota and Fusobacteriota were low (< 0.8%) and they are summarized as ‘Others’ in Figure 2A. Firmicutes (C: 49.9 ± 7.7%; HI25: 52.8 ± 7.5%; HI50: 43.4 ± 2.7%) was the dominant phylum in all three groups, followed by Bacteroidetes (C: 37.9 ± 8.4%; HI25: 34.2 ± 9.8%; HI50: 37.9 ± 4.3%). Regardless of the hens’ diet, the main orders of Firmicutes were the Lachnospirales represented by the family Lachnospiraceae and the Oscillospirales represented by the families Ruminococcaceae and Oscillospiraceae. Bacteroidetes was represented by Bacteroidales with the families Bacteroidaceae, Rikenellaceae and Prevotellaceae (Figure 2B). The less abundant phyla Proteobacteria, Actinobacteria, Cyanobacteria, Desulfobacterota and Campilobacterota were mainly represented by the families Sutterellaceae (class Gammaproteobacteria), Atopobiaceae (class Coriobacteriia), Gastranaerophilales (class Vampirivibrionia), Desulfovibrionaceae (class Desulfovibrionia) and Campylobacteraceae (class Campylobacteria), respectively. At the genus level, the most abundant genus was *Bacteroides*, followed by *Ruminococcus* torques group, *Faecalibacterium*, *Lactobacillus*, *Alistipes*, *Parasutterella* and *Christensenellaceae* R‐7 group, which together accounted for more than 46.6% of the sequences in each of the three groups of hens. The bacterial taxa with a relative abundance lower than 1% are summarized as ‘Others’ in Figure 2. The relative abundance (means ± SD) of bacterial taxa at different taxonomic levels is listed in Table S4, and the taxa with relative abundance lower than 0.5% are listed in Table S5.

### 3.3 Determination of Taxonomic Biomarkers

**FIGURE 3:**
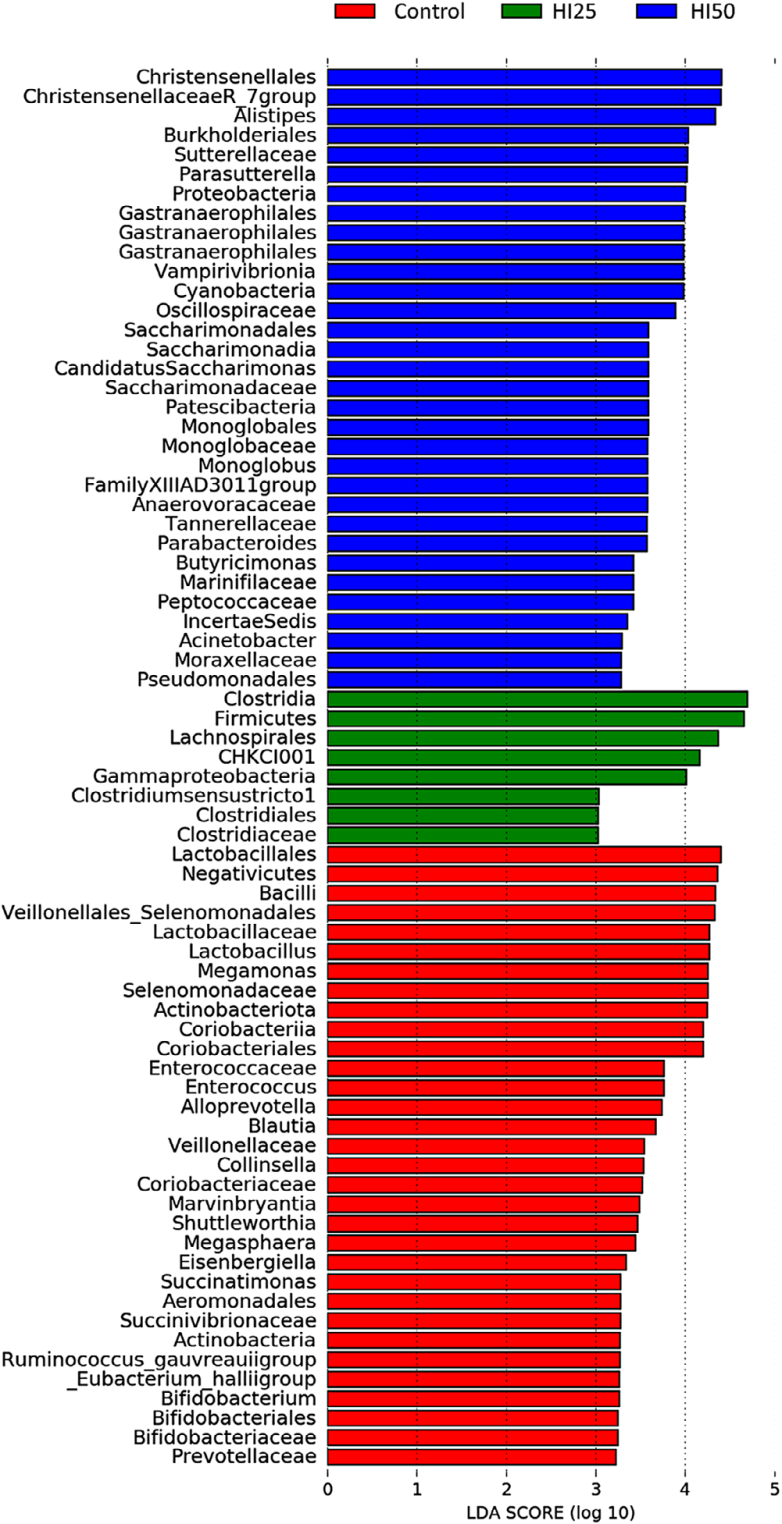
Histogram plots of linear discriminant analysis (LDA) scores on different taxonomical levels (phylum, class, order, family and genus) of the three groups of hens fed different diets (Control, HI25 and HI50). The length of the bar represents the log10 transformed LDA score, indicated by vertical dotted lines. Blue bars, green bars and red bars represent bacterial taxa over‐abundant in the corresponding group.

LEfSe was performed to determine the bacterial taxa that differed significantly in abundance among the control group and the groups of insect meal‐fed hens (Figure 3). The analysis comparing the three groups together resulted in 72 differentially abundant bacterial taxa (LDA score > 2.0). A total of 32 taxa had significantly higher relative abundance in the control group C (red bars), 8 taxa had significantly higher relative abundance in the HI25 group (green bars) and 32 taxa had significantly higher relative abundance in the HI50 group (blue bars).

In the control group, the increased class Bacilli (LDA score > 4.0) included the order Lactobacillales, the families Lactobacillaceae and Enterococcaceae, with the genera *Lactobacillus* and *Enterococcus*. The increased class Negativicutes (LDA score > 4.0) comprised the order Veillonellales–Selenomonadales, the families Selenomonadaceae and Veillonellaceae with the genera *Megamonas* and *Megasphaera*. The increased phylum Actinobacteriota (LDA score > 4.0) included the classes Actinobacteria and Coriobacteriia with several phylotypes of Coriobacteriales (Coriobacteriales, Coriobacteriaceae and *Collinsella*). The increased order Aeromonadales (LDA score > 3.0) comprised the family Succinivibrionaceae and the genus *Succinatimonas*. The increased order Bifidobacteriales (LDA score > 3.0) included the family Bifidobacteriaceae and the genus *Bifidobacterium*. The increased family Prevotellaceae included the genera *Alloprevotella*. The genera *Blautia*, *Marvinbryantia*, *Shuttleworthia*, *Eisenbergiella*, *Ruminococcus* gauvreauii group and *Eubacterium hallii* group had significantly higher relative abundance in the control group.

In the HI25 group, the increased phylum Firmicutes (LDA score > 4.0) included several phylotypes of Clostridiales (Clostridia, Clostridiales, Clostridiaceae and *Clostridium* sensu stricto 1). The increased order Lachnospirales included the genus *CHKCI001*. The class Gammaproteobacteria was also significantly more abundant in the HI25 group.

In the HI50 group, the increased phylum Proteobacteria (LDA score = 4.0) included the order Burkholderiales with family Sutterellaceae and genus *Parasutterella*. The increased phylum Cyanobacteria (LDA score = 4.0) included the class Vampirivibrionia and several phylotypes of Gastranaerophilales. The increased phylum Patescibacteria included several Saccharimonadia phylotypes (Saccharimonadales, Saccharimonadaceae and *Candidatus Saccharimonas*). The increased order Monoglobales (LDA score > 3.0) included the family Monoglobaceae with the genus *Monoglobus*. The increased order Pseudomonadales (LDA score > 3.0) included the family Moraxellaceae with the genus *Acinetobacter*. The increased order Christensenellales (LDA score > 4.0) included the genus *Christensenellaceae* R‐7 group. The families Oscillospiraceae, Anaerovoracaceae, Tannerellaceae, Marinifilaceae and Peptococcaceae had significantly higher relative abundance in the HI50 group. The genera *Alistipes*, Family XIII AD3011 group, *Parabacteroides*, *Butyricimonas* and *Incertae sedis* were also enriched in the HI50 group.

## 4 Discussion

The gastrointestinal microbiome of poultry plays a crucial role in nutrient digestion, absorption, growth performance and regulation of the immune system. A diet is known to be a major determinant of gut microbiota (Chen et al. 2023; X. Wei et al. 2023), thus changes in the dietary ingredients of poultry can modify the microbial community and affect the animal's physical condition, growth performance and productivity (Daniel 2022). It is now well known that a nutritional intervention intended to have a positive effect on the host should have a positive influence on the gut microbiome. From this point of view, the caecum is a particularly monitored section of the digestive tract, as it exhibits the highest bacterial count (10^11^ cells/g), bacterial diversity, long retention time of digesta and is considered the main site of fermentation processes (Elling‐Staats et al. 2022; Sergeant et al. 2014). Scientific research is mainly focused on broilers (Attia et al. 2023; Biasato et al. 2020; de Souza Vilela et al. 2021; Hayat et al. 2024), while there is much less information on hens (Elahi et al. 2022). Due to this knowledge gap, we specifically focused our study on the caecal microbiota of laying hens to assess potential diet‐induced microbial changes that are most important for nutrient utilization, gut health and productivity. By analysing this compartment, our results not only expand the current understanding of insect meal supplementation, but also provide new insights into the ecosystem of the caecum of laying hens, which has been little studied compared to broilers.

Our results show that insect meal used in the experiment had no significant effect on alpha bacterial diversity in the caeca of hens. Despite this finding, the comparison of two BSF meal doses indicates non‐significantly increased richness and evenness for 50% of BSF meal inclusion, while 25% of BSF supplementation non‐significantly increased only phylogenetic diversity. Nevertheless, our results are consistent with the study on broiler chickens by Vilela et al. (2023) and Colombino et al. (2021), who did not find significant differences in alpha indices. On the other hand, our outcomes are in contradiction with the findings of studies on hens by Yan et al. (2023), Borrelli et al. (2017) and Kawasaki et al. (2019), who reported a higher bacterial richness in the treatment groups compared to the control group. Kawasaki et al. (2019), however, described a higher Chao1 index, although consistent with our findings, they did not observe any differences in Shannon diversity. However, the significant impact of BSF larvae meal on caecal bacteria in our experiment is documented by the spatial separation of the samples determined by PCoA based on the Jaccard distance matrix, and this finding is in agreement with the results of the studies of Yan et al. (2023) and Borrelli et al. (2017) on hens, but in contradiction with the studies of de Souza Vilela et al. (2023) and Colombino et al. (2021) on broilers. It can be assumed that the inconsistency of results is related to the different amounts of insect meal in the diet, but the genotype of poultry and the type of experiment can also play a role.

The bacterial community composition in the caecal samples of all three groups of hens was dominated by Firmicutes, followed by Bacteroidetes, the two most abundant phyla known to prevail in the caecum of poultry. The prevalence of Firmicutes has been described in caecal samples of laying hens regardless of the type of diet (Birkl et al. 2018; Borrelli et al. 2017; Joat et al. 2021; Ndotono et al. 2022), which is in agreement with our study. However, other studies found Bacteroidetes as the dominant phylum (Adhikari et al. 2020; Chang et al. 2022; Huang et al. 2019; Xing et al. 2019). These changes may be associated with the different physiological stages of the laying period. Indeed, the early period of hen life is characterized by the prevalence of the phylum Proteobacteria. Later, the dominance of Firmicutes is observed and consequently the relative abundance of Bacteroidetes increases, Firmicutes decreases and the balance of these two phyla is usually established during the peak production period. In the late phase, Bacteroidetes are more abundant than Firmicutes (Dai et al. 2022).

At the family level, Bacteroidaceae, Ruminococcaceae, Lachnospiraceae and Oscillospiraceae were the dominant families in all three groups, together accounting for more than half of the sequences, which is in agreement with several studies (Dai et al. 2022; Joat et al. 2021; Lundberg et al. 2021). Strains of the Bacteroidaceae family are thought to be involved in practically all bacterial functional gene categories in the gut microbiome (Yap et al. 2014). Although they do not produce butyrate, it is known from studies on humans that they can promote the butyrate pool through their acetate production, which can be involved as a co‐substrate in the butyryl‐CoA transferase pathway in the gut bacteria (Poeker et al. 2018). Ruminococcaceae and Lachnospiraceae are butyrate producers and play an important role in the maintenance of gastrointestinal health (Medvecky et al. 2018; Rychlik 2020). Oscillospiraceae is also a poultry‐dominant family producing butyrate (Segura‐Wang et al. 2021). Strains of this family are thought to have anti‐inflammatory properties and are linked to health markers (Meslier et al. 2022). The *Oscillospira* species have already been even suggested as one of the next‐generation probiotic candidates (Yang et al. 2021). The interesting recent research of Volf et al. (2024) revealed that members of Lachnospiraceae, Ruminococcaceae and Oscillospiraceae families require expression of immunoglobulins for their colonisation of the chicken caecum. The coating of these bacteria by immunoglobulins probably contributes to the stabilisation of the caecal microbiota and is also important for the elimination of unwanted microbes.

At the genus level, sequences of *Bacteroides* were largely dominating in the caeca of all three groups of hens. The predominance of this taxon in the caecum of laying hens has already been described in several studies (Adhikari et al. 2020; Huang et al. 2019; Xing et al. 2019), and it can be considered beneficial. Experiments performed with a defined mixture of several *Bacteroides* strains indicate their protective role in chicken caeca associated with reduced colonization by pathogenic bacteria (Papouskova et al. 2023). Other detected taxa, such as *Faecalibacterium*, *Lactobacillus*, *Alistipes*, *Parasutterella* and *Christensenellaceae* R‐7 group, are also known to be a beneficial and important part of the caecal microbiome of poultry (Atallah et al. 2023, 2025; Biasato et al. 2020; Cardenas et al. 2021; Mahayri et al. 2024; Polansky et al. 2016).

The influence of *H. illucens* larvae was clearly observed at all taxonomic levels. LEfSE analysis revealed 72 differentially abundant bacterial taxa among the groups. Several beneficial genera were enriched in the control group, including *Lactobacillus*, *Megamonas, Bifidobacterium*, *Blautia* and *Ruminococcus* gauvreauii group. This is in agreement with the study of Kawasaki et al. (2019), who found a significantly lower relative abundance of *Lactobacillus* and *Bifidobacterium* populations in the treatment groups compared to the control group, as well as the study of He et al. (2021), who revealed a higher relative abundance of *Megamonas* in the group of chickens fed the basal diet compared to the groups fed *H. illucens* larvae meal. On the other hand, *Enterococcacae* family and *Enterococcus* genus, which can act as opportunistic pathogens and virulent species associated with enterococcal diseases in poultry (Alzahrani et al. 2022; Souillard et al. 2022), were significantly more abundant in the control group, which is in agreement with the study of Vilela et al. (2023). The suppression of these taxa in the insect meal‐fed hens, therefore, may be regarded as beneficial. Coriobacteriales phylotypes enriched in the control group can also include opportunistic pathobionts associated with several infections in humans and animals, such as periodontitis, bacteremia and vaginosis (Clavel et al. 2014; Ramasamy et al. 2014). On the other hand, members of this taxon provide important functions, such as the conversion of bile salts and steroids or the activation of dietary polyphenols, so the evaluation of their role is problematic and they cannot be clearly assessed as negative components of the microbiome. *Collinsella* (Coriobacteriaceae family), enriched in the control group, is commonly detected in the caecum of adult hens (Rychlik et al. 2023); its function is unknown, but it is not directly associated with any disease of poultry. In the HI25 group, the phylum Firmicutes was significantly more abundant, including several enriched phylotypes of Clostridiales, among which were the beneficial Lachnospirales (Liu et al. 2021). However, compared to the HI50 group, the effect of the insect meal on HI25 group was lower.

Several bacterial genera enriched by BSF meal in the HI50 group, including *Parasutterella*, *Parabacteroides, Butyricimonas*, *Christensenellaceae* R‐7 group, *Family* XIII AD3011 group and *Alistipes* can be considered beneficial. The increase of the latter three taxa was also observed by Yan et al. (2023), despite their use of much lower doses of *H. illucens* larvae meal (1%–3%) in the diet of hens. The *Christensenellaceae* R‐7 group, *Alistipes* and *Butyricimonas* produce short‐chain fatty acids, including butyric acid, which is important for supporting epithelial barrier function (Morotomi et al. 2011). Clostridial family XIII AD3011 contributes to protein breakdown (Polansky et al. 2016). *Parasutterella* is supposed to contribute to host, health and its potential role consists of maintaining bile acid homeostasis and cholesterol metabolism (Ju et al. 2019). *Parabacteroides* seems to be positively correlated with broiler body weight (Marcolla et al. 2023). These two genera have been described as part of the core microbiota of broilers reared in extensive production systems (Marcolla et al. 2023).

## 5 Conclusion

This study has shown that the inclusion of *H. illucens* larval meal in the diet of laying hens may have an impact on the bacterial composition of the caecum, leading to changes in the relative abundance of some bacterial taxa, with a decrease in some opportunistic pathogens. The bacterial richness and evenness were similar; however, the beta diversity revealed significant dissimilarities among the groups of hens. The significant increase in some beneficial bacterial taxa indicates a possible positive effect of used insect meal, with 50% replacement, showing the greatest impact. However, the functional implications of these changes remain to be explored, and it is unclear how different inclusion levels might affect long‐term hens’ health and microbiome. Further investigations examining a wider range of BSFL inclusion rates and linking microbial shifts to physiological and productive outcomes will help to clarify the practical potential of insect‐based diets in poultry.

## Author Contributions

Conceptualization: Kateřina Olša Fliegerová, Fulvia Bovera, Giuseppe Moniello and Giovanni Piccolo. Methodology: Kateřina Olša Fliegerová, Jakub Mrázek, Tiziana Maria Mahayri and Elie Atallah. Formal Analysis and Investigation: Tiziana Maria Mahayri and Elie Atallah. Resources: Fulvia Bovera and Giuseppe Moniello. Data Curation: Tiziana Maria Mahayri and Elie Atallah. Writing – Original Draft Preparation: Tiziana Maria Mahayri. Writing – Review and Editing: Kateřina Olša Fliegerová, Fulvia Bovera, Giuseppe Moniello and Giovanni Piccolo. All authors have read and agreed to the published version of the manuscript.

## Conflicts of Interest

The authors declare no conflicts of interest.

## Peer Review

The peer review history for this article is available at https://www.webofscience.com/api/gateway/wos/peer‐review/10.1002/vms3.70650.

## Supporting information




**Table S1**. Proximate composition, mineral and essential amino acid composition (% as fed) of the *Hermetia illucens* larvae meal and soybean meal.
**Table S2**. Ingredients and chemical‐nutritional characteristics of diets.
**Table S3**. Animals’ characteristics and samples weight for DNA extraction.
**Table S4**. Relative abundance (means ± SD) of bacterial taxa at phylum, class, order, family and genus levels.
**Figure S1**. Rarefaction curves representing the sequencing depth (number of reads) and the number of ASVs (sequence variants) found in the cecal content of hens.

## Data Availability

The data presented in this study are openly available in the Sequence Read Archive under accession number: PRJNA962558.References
