# Supplementary material for: The Inclusion of Hermetia Illucens Larvae Meal in the Diet of Laying Hens (Hy‐Line Brown) Affects the Caecal Bacterial Composition and Diversity
Source: Vet Med Sci. 2025 Oct 21;11(6):e70650. doi: 10.1002/vms3.70650 (PMC12538631; doi:10.1002/vms3.70650)
Supplement: Supplementary file 1 — Table S1. Proximate composition, mineral and essential amino acid composition (% as fed) of the Hermetia illucens larvae meal and soybean meal. Table S2. Ingredients and chemical‐nutritional characteristics of diets. Table S3. Animals’ characteristics and samples weight for DNA extraction. Table S4. Relative abundance (means ± SD) of bacterial taxa at phylum, class, order, family and genus levels. Figure S1. Rarefaction curves representing the sequencing depth (number of reads) and the number of ASVs (sequence variants) found in the cecal content of hens. [file VMS3-11-e70650-s001.docx]

**Supplementary Material**

**The inclusion of *Hermetia illucens* larvae meal in the diet of laying hens (Hy-line Brown) affects the caecal bacterial composition and diversity**

Tiziana Maria Mahayri ^1, 2^, Elie Atallah ^2, 4^, Jakub Mrázek ^1^, Fulvia Bovera ^3^, Giovanni Piccolo ^3^, Giuseppe Moniello ^2,*^, Kateřina Olša Fliegerová ^1^

^1^ Laboratory of Anaerobic Microbiology, Institute of Animal Physiology and Genetics, Czech Academy of Science, 14220 Prague, Czech Republic

^2^ Department of Veterinary Medicine, University of Sassari, Via Vienna, 2, 07100 Sassari, Italy

^3^ Department of Veterinary Medicine and Animal Production, University of Napoli Federico II, Via F. Delpino, 1, 80137 Napoli, Italy

^4^  Department of Veterinary Medicine and Animal Sciences, University of Milan, Via dell’Università, 6, Lodi 26900, Italy

* Corresponding author. Tel.: +39 079 229443; EM: moniello@uniss.it

**Table S1.** Proximate composition, mineral and essential amino acid composition (% as fed) of the *Hermetia illucens* larvae meal and soybean meal.

|  | ***Hermetia illucens* Larvae meal** | **Soybean meal** |
| --- | --- | --- |
| **Proximate composition** | | |
| Dry matter | 92.7 | 90.0 |
| Crude protein | 55.6 | 43.4 |
| Ether extract | 8.34 | 1.1 |
| ADF | 11.5 | 5.9 |
| ADF-linked protein | 4.86 | 1.78 |
| Ash | 7.8 | 6.0 |
| **Mineral composition** | | |
| Ca^a^ | 6.47 | 2.83 |
| Total P^a^ | 0.90 | 0.57 |
| Na^a^ | 0.12 | 0.16 |
| **Essential amino acid composition** | | |
| Lysine^a^ | 4.12 | 2.92 |
| Methionine^a^ | 1.09 | 0.61 |
| Methionine + Cystine^a^ | 1.32 | 1.33 |
| Isoleucine^a^ | 2.97 | 2.30 |
| Tryptophan^a^ | 0.30 | 0.73 |
| Valine^a^ | 5.02 | 2.11 |
| Threonine^a^ | 2.32 | 1.74 |

^a^ Obtained by manufacturing company.

**Table S2.** Ingredients and chemical-nutritional characteristics of diets.

|  | **C** | **HI25** | **HI50** |
| --- | --- | --- | --- |
| **Ingredients, g/kg** | | | |
| Maize grain | 606 | 598 | 631 |
| Soybean meal | 265 | 200 | 95 |
| Insect meal | – | 73 | 146 |
| CaCO_3_ grains | 80 | 80 | 80 |
| Vegetable oil | 10 | 10 | – |
| MinVit^c^ | 10 | 10 | 10 |
| Methionine | 2.5 | 2.0 | 2.0 |
| Monocalcium phosphate | 5 | 5 | 5 |
| Celite | 20 | 20 | 20 |
| Salt | 2 | 2 | 2 |
| **Chemical-nutritional characteristics** | | | |
| Dry matter,^a^ % | 91.53 | 91.39 | 91.62 |
| Crude protein,^a^ % | 16.45 | 16.32 | 17.03 |
| Ether extract,^a^ % | 3.37 | 3.51 | 3.86 |
| NDF,^a^ % | 10.38 | 11.29 | 12.49 |
| ADF,^a^ % | 5.85 | 5.90 | 5.67 |
| ADL,^a^ % | 2.67 | 2.94 | 2.29 |
| Lysine,^b^ % | 0.90 | 0.97 | 1.00 |
| Methionine,^b^ % | 0.53 | 0.54 | 0.54 |
| Metabolizable Energy,^b^ kcal/kg | 2832.3 | 2845.2 | 2842.2 |

C: soybean meal based diet; HI25: diet including *Hermetia illucens* as 25% of replacement of the soybean meal protein; HI50: diet including *Hermetia illucens* as 50% of replacement of the soybean meal protein;

^a^ Determined according to AOAC (2005)

^b^ Calculated according to NRC (1994)

^c^ Provided per kilogram: vitamin A (retinyl acetate) 20,000 IU, vitamin D3 (cholecalciferol) 6000 IU, vitamin E (dl-α-tocopheryl acetate) 80 IU, vitamin B1(thiamine monophosphate) 3 mg, vitamin B2 (riboflavin) 12 mg, vitamin B6 (pyridoxine hydrochloride) 8 mg, vitamin B12 (cyanocobalamin) 0.04 mg, vitamin K3 (menadione) 4.8 mg, vitamin H (d biotin) 0.2 mg, vitamin PP (nicotinic acid) 48 mg, folic acid 2 mg, calcium pantothenate 20 mg, manganous oxide 200 mg, ferrous carbonate 80 mg, cupric sulphate pentahydrate 20 mg, zinc oxide 120 mg, basic carbonate monohydrate 0.4 mg, anhydrous calcium iodate 2 mg, sodium selenite 0.4 mg, choline chloride 800 mg, 4–6-phitase 1800 FYT, D.L. methionine 2600 mg, canthaxanthin 8 mg.

**Table S3.** Animals’ characteristics and samples weight for DNA extraction.

| **Sample name** | **Diet** | **Age** | **Weight for DNA extraction** |
| --- | --- | --- | --- |
| H1-2 | Control | 40 weeks | 80mg |
| H1-3 | Control | 40 weeks | 79mg |
| H1-4 | Control | 40 weeks | 79mg |
| H1-5 | Control | 40 weeks | 83mg |
| H1-6 | Control | 40 weeks | 78mg |
| H1-7 | Control | 40 weeks | 80mg |
| H1-8 | Control | 40 weeks | 79mg |
| H1-9 | Control | 40 weeks | 81mg |
| H1-10 | Control | 40 weeks | 80mg |
| H2-1 | H50 | 40 weeks | 93mg |
| H2-2 | H50 | 40 weeks | 136mg |
| H2-3 | H50 | 40 weeks | 101mg |
| H2-5 | H50 | 40 weeks | 112mg |
| H2-6 | H50 | 40 weeks | 145mg |
| H2-7 | H50 | 40 weeks | 118mg |
| H2-8 | H50 | 40 weeks | 140mg |
| H2-9 | H50 | 40 weeks | 118mg |
| H2-10 | H50 | 40 weeks | 105mg |
| H3-1 | H25 | 40 weeks | 90mg |
| H3-2 | H25 | 40 weeks | 81mg |
| H3-3 | H25 | 40 weeks | 102mg |
| H3-4 | H25 | 40 weeks | 88mg |
| H3-5 | H25 | 40 weeks | 99mg |
| H3-6 | H25 | 40 weeks | 101mg |
| H3-7 | H25 | 40 weeks | 87mg |
| H3-8 | H25 | 40 weeks | 85mg |
| H3-9 | H25 | 40 weeks | 80mg |

**Table S4.** Relative abundance (means ± SD) of bacterial taxa at phylum, class, order, family and genus levels.

| **Taxonomic levels** | **Control** | **H25** | **H50** |
| --- | --- | --- | --- |
| **Phylum** |  |  |  |
| Firmicutes | 49.9 ± 7.7% | 52.8 ± 7.5% | 43.4 ± 2.7% |
| Bacteroidetes | 37.9 ± 8.4% | 34.2 ± 9.8% | 37.9 ± 4.3% |
| Verrucomicrobiota | 0.1 ± 0.1% | 0.2 ± 0.2% | 0.8 ± 0.4% |
| Proteobacteria | 3.6 ± 1% | 5 ± 1.6% | 7 ± 1.6% |
| Spirochaetota | 0.03 ± 0.1% | 0.9 ± 0.9% | 0.3 ± 0.6% |
| Patescibacteria | 0.2 ± 0.4% | 0.2 ± 0.5% | 0.5 ± 1.3% |
| Actinobacteriota | 5 ± 3.8% | 3.3 ± 2.4% | 2.9 ± 1.1% |
| Synergistota | 0.3 ± 0.2% | 0.3 ± 0.5% | 0.7 ± 0.3% |
| Elusimicrobiota | 0.001 ± 0.002% | 0.1 ± 0.1% | 0.1 ± 0.2% |
| Desulfobacterota | 1.2 ± 0.6% | 1.2 ± 0.6% | 1.3 ± 0.4% |
| Cyanobacteria | 0.03 ± 0.4% | 0.3 ± 0.5% | 3.8 ± 1.9% |
| Fusobacteriota | 0.3 ± 1.3% | 0.7 ± 2.3% | 0.06 ± 0.2% |
| Campilobacterota | 1.1 ± 0.7% | 0.4 ± 0.5% | 0.8 ± 0.4% |
| Deferribacterota | 0.1 ± 0.1% | 0.2 ± 0.4% | 0.4 ± 0.2% |
| **Class** |  |  |  |
| Clostridia | 36.6 ± 7.8% | 46.2 ± 7.3% | 40.5 ± 2.4% |
| Campylobacteria | 1.1 ± 0.7% | 0.4 ± 0.5% | 0.8 ± 0.4% |
| Negativicutes | 6.2 ± 3.1% | 2.8 ± 2% | 1.3 ± 0.5% |
| Bacteroidia | 37.9 ± 8.5% | 34.2 ± 9.8% | 37.9 ± 4.3% |
| Lentisphaeria | 0.1 ± 0.1% | 0.2 ± 0.2% | 0.8 ± 0.4% |
| Gammaproteobacteria | 3.6 ± 0.8% | 5 ± 1.6% | 6.6 ± 1.5% |
| Bacilli | 6.9 ± 2.4% | 3.7 ± 3.1% | 1.5 ± 1.6% |
| Brachyspirae | 0.03 ± 0.1% | 0.8 ± 0.9% | 0.2 ± 0.09% |
| Coriobacteriia | 4.5 ± 3.7% | 3.2 ± 2.2% | 2.9 ± 1.1% |
| Synergistia | 0.3 ± 0.2% | 0.3 ± 0.5% | 0.7 ± 0.3% |
| Desulfovibrionia | 1.2 ± 0.6% | 1.2 ± 0.6% | 1.3 ± 0.4% |
| Vampirivibrionia | 0.03 ± 0.4% | 0.3 ± 0.5% | 3.8 ± 1.9% |
| Fusobacteriia | 0.3 ± 1.3% | 0.7 ± 2.3% | 0.06 ± 0.2% |
| **Order** |  |  |  |
| Oscillospirales | 10.4 ± 1.9% | 15 ± 3.7% | 17.5 ± 1.5% |
| Campylobacterales | 1.1 ± 0.7% | 0.4 ± 0.5% | 0.8 ± 0.4% |
| Acidaminococcales | 1.2 ± 0.4% | 1 ± 0.3% | 1.1 ± 0.5% |
| Lachnospirales | 19.5 ± 4.6% | 19.7 ± 4.7% | 12.8 ± 3.6% |
| Bacteroidales | 37.9 ± 8.5% | 34.2 ± 9.6% | 37.9 ± 4.3% |
| Christensenellales | 0.5 ± 0.3% | 3.9 ± 1.7% | 4.4 ± 1.9% |
| Victivallales | 0.1 ± 0.1% | 0.2 ± 0.2% | 0.8 ± 0.4% |
| Burkholderiales | 3 ± 0.9% | 4.6 ± 1.4% | 6.4 ± 1.6% |
| Lactobacillales | 5.9 ± 2.1% | 2.3 ± 2.2% | 0.7 ± 0.8% |
| Brachyspirales | 0.03 ± 0.1% | 0.8 ± 0.9% | 0.2 ± 0.09% |
| RF39 | 0.4 ± 0.4% | 0.8 ± 0.5% | 0.5 ± 1.5% |
| Clostridia UCG-014 | 1.1 ± 0.6% | 1 ± 0.5% | 0.8 ± 1.9% |
| Clostridia vadinBB60 group | 2.4 ± 2% | 4 ± 2.4% | 1.8 ± 1.3% |
| Coriobacteriales | 4.5 ± 3.7% | 3.2 ± 2.2% | 2.9 ± 1.1% |
| Erysipelotrichales | 0.5 ± 0.2% | 0.7 ± 1.1% | 0.3 ± 0.3% |
| Synergistales | 0.3 ± 0.2% | 0.3 ± 0.5% | 0.7 ± 0.3% |
| Veillonellales-Selenomonadales | 5 ± 3.2% | 1.8 ± 2% | 0.3 ± 0.2% |
| Desulfovibrionales | 1.2 ± 0.6% | 1.2 ± 0.6% | 1.3 ± 0.4% |
| Aeromonadales | 0.6 ± 0.3% | 0.3 ± 0.3% | 0.1 ± 0.09% |
| Peptostreptococcales-Tissierellales | 2.4 ± 1.6% | 2 ± 2.5% | 2.1 ± 1.7% |
| Gastranaerophilales | 0.03 ± 0.4% | 0.3 ± 0.5% | 3.8 ± 1.9% |
| Fusobacteriales | 0.3 ± 1.3% | 0.7 ± 2.3% | 0.06 ± 0.2% |
| **Family** |  |  |  |
| Ruminococcaceae | 8.1 ± 1.9% | 9.2 ± 3.1% | 8.5 ± 2.2% |
| Campylobacteraceae | 1 ± 0.8% | 0.3 ± 0.4% | 0.8 ± 0.4% |
| Acidaminococcaceae | 1.2 ± 0.4% | 1 ± 0.3% | 1.1 ± 0.5% |
| Lachnospiraceae | 19.5 ± 4.6% | 19.7 ± 4.7% | 12.8 ± 3.6% |
| Rikenellaceae | 5.4 ± 2.3% | 5 ± 5.7% | 6.5 ± 4% |
| Christensenellaceae | 0.6 ± 0.3% | 3.9 ± 1.7% | 4.4 ± 1.9% |
| Victivallaceae | 0.1 ± 0.1% | 0.2 ± 0.2% | 0.8 ± 0.4% |
| Tannerellaceae | 0.6 ± 0.4% | 1.1 ± 0.5% | 1.2 ± 0.6% |
| Oscillospiraceae | 1.6 ± 0.5% | 4.4 ± 1.8% | 7.5 ± 1.8% |
| Bacteroidaceae | 27 ± 6.5% | 24.5 ± 6.3% | 27 ± 4.6% |
| Prevotellaceae | 2.3 ± 1.1% | 1.3 ± 0.9% | 0.9 ± 1.4% |
| Lactobacillaceae | 4.8 ± 2.2% | 2.1 ± 1.9% | 0.6 ± 0.8% |
| Brachyspiraceae | 0.03 ± 0.1% | 0.8 ± 0.9% | 0.2 ± 0.09% |
| Sutterellaceae | 3 ± 0.9% | 4.6 ± 1.5% | 6.3 ± 1.6% |
| RF39 | 0.4 ± 0.4% | 0.8 ± 0.5% | 0.5 ± 1.5% |
| Clostridia UCG-014 | 1.1 ± 0.6% | 1 ± 0.5% | 0.8 ± 1.9% |
| Clostridia vadinBB60 group | 2.4 ± 2% | 4 ± 2.4% | 1.8 ± 1.3% |
| Butyricicoccaceae | 0.4 ± 0.7% | 1.1 ± 0.8% | 0.7 ± 0.3% |
| Unclassified family within order Bacteroidales | 1.2 ± 0.8% | 0.8 ± 0.4% | 0.9 ± 1% |
| Erysipelotrichaceae | 0.4 ± 0.3% | 0.6 ± 1% | 0.2 ± 0.3% |
| Synergistaceae | 0.3 ± 0.2% | 0.3 ± 0.5% | 0.7 ± 0.3% |
| Atopobiaceae | 3.5 ± 3.4% | 2.5 ± 1.5% | 2.5 ± 0.9% |
| Coriobacteriaceae | 0.9 ± 0.3% | 0.6 ± 0.8% | 0.3 ± 0.2% |
| Veillonellaceae | 0.9 ± 0.6% | 0.3 ± 0.7% | 0.02 ± 0.05% |
| Desulfovibrionaceae | 1.2 ± 0.6% | 1.2 ± 0.6% | 1.3 ± 0.4% |
| Succinivibrionaceae | 0.6 ± 0.3% | 0.3 ± 0.3% | 0.1 ± 0.09% |
| Unclassified family within order Bacteroidales | 0.9 ± 0.3% | 0.8 ± 2.4% | 1.2 ± 0.6% |
| Selenomonadaceae | 4.2 ± 2.6% | 1.5 ± 1.9% | 0.2 ± 0.2% |
| Barnesiellaceae | 0.2 ± 0.3% | 0.5 ± 0.5% | 0.1 ± 0.2% |
| Anaerovoracaceae | 0.2 ± 0.1% | 0.6 ± 1% | 0.8 ± 1.2% |
| Peptostreptococcaceae | 2.2 ± 1.5% | 1.5 ± 2.4% | 1.3 ± 1% |
| Gastranaerophilales | 0.03 ± 0.4% | 0.3 ± 0.5% | 3.8 ± 1.9% |
| Fusobacteriaceae | 0.3 ± 1.2% | 0.7 ± 2.3% | 0.1 ± 0.2% |
| Enterococcaceae | 1 ± 0.9% | 0.2 ± 0.5% | 0.1 ± 0.05% |
| **Genus** |  |  |  |
| Campylobacter | 1 ± 0.8% | 0.3 ± 0.4% | 0.8 ± 0.4% |
| Phascolarctobacterium | 1.2 ± 0.4% | 1 ± 0.3% | 1.1 ± 0.5% |
| Ruminococcus torques group | 8.7 ± 2.2% | 7.1 ± 2.4% | 5.8 ± 3% |
| Negativibacillus | 0.7 ± 0.5% | 0.5 ± 0.7% | 0.6 ± 0.3% |
| Alistipes | 2.3 ± 1.2% | 1.9 ± 1.6% | 5.3 ± 3.3% |
| Christensenellaceae R-7 group | 0.6 ± 0.3% | 3.9 ± 1.7% | 4.2 ± 1.8% |
| Unclassified genus within family Ruminococcaceae | 0.5 ± 0.3% | 0.9 ± 0.8% | 1 ± 0.6% |
| Parabacteroides | 0.6 ± 0.4% | 1.1 ± 0.5% | 1.2 ± 0.6% |
| Unclassified genus within family Lachnospiraceae | 2.8 ± 0.9% | 4 ± 1.4% | 2.6 ± 1.9% |
| Oscillibacter | 0.3 ± 0.2% | 0.6 ± 0.6% | 0.9 ± 1.1% |
| Bacteroides | 27 ± 6.5% | 24.5 ± 6.3% | 27 ± 4.6% |
| Prevotellaceae UCG-001 | 0.6 ± 0.3% | 0.3 ± 0.2% | 0.3 ± 0.4% |
| Shuttleworthia | 0.9 ± 0.3% | 0.7 ± 0.3% | 0.3 ± 0.7% |
| Lactobacillus | 4.8 ± 2.2% | 2.1 ± 1.9% | 0.6 ± 0.8% |
| Brachyspira | 0.03 ± 0.1% | 0.8 ± 0.9% | 0.2 ± 0.09% |
| Unclassified genus within family Oscillospiraceae | 0.5 ± 0.3% | 1.3 ± 0.8% | 1.3 ± 1.4% |
| Sutterella | 1.3 ± 0.5% | 1.6 ± 1.1% | 1.4 ± 0.8% |
| RF39 | 0.4 ± 0.4% | 0.8 ± 0.5% | 0.5 ± 1.5% |
| Clostridia UCG-014 | 1.1 ± 0.6% | 1 ± 0.5% | 0.8 ± 1.9% |
| Clostridia vadinBB60 group | 2.4 ± 2% | 4 ± 2.4% | 1.8 ± 1.3% |
| Parasutterella | 1.7 ± 0.8% | 3 ± 1.6% | 5 ± 1.3% |
| Butyricicoccus | 0.3 ± 0.2% | 0.9 ± 0.6% | 0.4 ± 0.2% |
| Oscillospiraceae UCG-005 | 0.04 ± 0.06% | 1.3 ± 1.1% | 0.7 ± 0.8% |
| Paraprevotella | 0.3 ± 0.3% | 0.7 ± 0.9% | 0.2 ± 1.2% |
| Unclassified genus within order Bacteroidales | 1.2 ± 0.8% | 0.8 ± 0.4% | 0.9 ± 1% |
| Blautia | 1 ± 0.4% | 0.9 ± 0.4% | 0.3 ± 0.1% |
| Unclassified genus within family Oscillospiraceae | 0.4 ± 0.3% | 0.5 ± 0.6% | 2 ± 1% |
| Faecalibacterium | 4.3 ± 1.2% | 4.3 ± 2.6% | 3.4 ± 1.1% |
| Synergistes | 0.3 ± 0.2% | 0.3 ± 0.5% | 0.7 ± 0.3% |
| Rikenellaceae RC9 gut group | 3.1 ± 1.3% | 3.1 ± 5% | 1.2 ± 2.3% |
| Olsenella | 3.5 ± 3.4% | 2.5 ± 1.5% | 2.5 ± 0.8% |
| Subdoligranulum | 1.4 ± 0.4% | 1.5 ± 1.2% | 0.9 ± 0.7% |
| Oscillospiraceae NK4A214 group | 0.04 ± 0.3% | 0.5 ± 0.5% | 1.8 ± 1% |
| Megasphaera | 0.7 ± 0.4% | 0.3 ± 0.7% | 0 |
| Desulfovibrio | 1.1 ± 0.5% | 1.1 ± 0.5% | 0.9 ± 0.3% |
| Succinatimonas | 0.6 ± 0.3% | 0.3 ± 0.3% | 0.1 ± 0.09% |
| Collinsella | 0.9 ± 0.3% | 0.5 ± 0.7% | 0.3 ± 0.2% |
| Unclassified genus within family Ruminococcaceae | 0.6 ± 0.5% | 0.7 ± 0.5% | 0.5 ± 0.4% |
| Unclassified genus within order Bacteroidales | 0.9 ± 0.3% | 0.8 ± 2.4% | 1.2 ± 0.6% |
| Megamonas | 4.2 ± 2.6% | 1.5 ± 1.9% | 0.2 ± 0.2% |
| Eisenbergiella | 0.5 ± 0.2% | 0.3 ± 0.2% | 0.4 ± 0.2% |
| Ruminococcus | 0.05 ± 0.08% | 0.8 ± 0.7% | 1.4 ± 0.9% |
| Family XIII AD3011 group | 0.2 ± 0.1% | 0.5 ± 1% | 0.8 ± 1.2% |
| Lachnoclostridium | 1.3 ± 0.3% | 1.6 ± 0.6% | 1.7 ± 0.5% |
| Lachnospiraceae GCA-900066575 | 0.5 ± 0.2% | 0.6 ± 0.4% | 0.6 ± 0.3% |
| Victivallaceae | 0.1 ± 0.08% | 0.1 ± 0.1% | 0.6 ± 0.3% |
| Romboutsia | 2.2 ± 1.5% | 1.5 ± 2.4% | 1.3 ± 1% |
| Gastranaerophilales | 0.03 ± 0.4% | 0.3 ± 0.5% | 3.8 ± 1.9% |
| Fusobacterium | 0.3 ± 1.3% | 0.7 ± 2.3% | 0.1 ± 0.2% |
| CHKCI001 | 2.2 ± 1% | 3.4 ± 2.3% | 0.1 ± 0.1% |
| Alloprevotella | 1.1 ± 0.8% | 0.3 ± 0.2% | 0.1 ± 0.3% |
| Enterococcus | 1 ± 0.9% | 0.2 ± 0.5% | 0.1 ± 0.05% |

**Table S5.** Bacterial classes, orders, families and genera with low relative abundance (< 0.5%).

| **Bacterial Class** | **Control** | **H25** | **H50** |
| --- | --- | --- | --- |
| Deferribacteres | 0.125751 | 0.198227 | 0.379517 |
| Saccharimonadia | 0.257490169 | 0.211590717 | 0.487397375 |
| Spirochaetia | 0 | 0.09354537 | 0.1228175 |
| Elusimicrobia | 0.001198 | 0.098 | 0.099029 |
| Incertae Sedis | 0 | 0.017818 | 0.038726 |
| Alphaproteobacteria | 0.049901 | 0 | 0.395561 |
| Actinobacteria | 0.423561 | 0.140318 | 0.034854 |
| **Bacterial Order** |  |  |  |
| Deferribacterales | 0.125751 | 0.198227 | 0.379517 |
| Saccharimonadales | 0.25749 | 0.211591 | 0.487397 |
| Pseudomonadales | 0.001996 | 0.017818 | 0.07358 |
| Spirochaetales | 0 | 0.093545 | 0.122817 |
| Elusimicrobiales | 0.001198 | 0.098 | 0.099029 |
| DTU014 | 0 | 0.017818 | 0.038726 |
| Monoglobales | 0.03034 | 0.224954 | 0.424329 |
| Enterobacterales | 0 | 0.0735 | 0.008852 |
| Rhodospirillales | 0.049901 | 0 | 0.395561 |
| Peptococcales | 0.204795 | 0.300682 | 0.490164 |
| Corynebacteriales | 0.012375 | 0.004455 | 0 |
| Clostridiales | 0.049103 | 0.064591 | 0.002766 |
| Pasteurellales | 0.001597 | 0.004455 | 0.002766 |
| Bifidobacteriales | 0.409589 | 0.135864 | 0.032087 |
| Micrococcales | 0.001597 | 0 | 0.002766 |
| Clostridia | 0 | 0 | 0.014384 |
| Flavobacteriales | 0.000798 | 0.062364 | 0.036513 |
| Eubacteriales | 0 | 0.004455 | 0.008852 |
| Staphylococcales | 0.002794 | 0 | 0 |
| **Bacterial family** |  |  |  |
| Deferribacteraceae | 0.125751 | 0.198227 | 0.379517 |
| Oxalobacteraceae | 0.012375 | 0.022273 | 0.088517 |
| Marinifilaceae | 0.027545 | 0.095773 | 0.099582 |
| Saccharimonadaceae | 0.25749 | 0.211591 | 0.487397 |
| Oscillospirales UCG-010 | 0.148506 | 0.2205 | 0.494036 |
| Unclassified family within order Oscillospirales | 0.09102 | 0.133636 | 0.188652 |
| Muribaculaceae | 0.282241 | 0.089091 | 0.044259 |
| Moraxellaceae | 0.001996 | 0.017818 | 0.07358 |
| Eggerthellaceae | 0.08064 | 0.055682 | 0.075793 |
| Spirochaetaceae | 0 | 0.093545 | 0.122817 |
| Elusimicrobiaceae | 0.001198 | 0.098 | 0.099029 |
| DTU014 | 0 | 0.017818 | 0.038726 |
| Monoglobaceae | 0.03034 | 0.224954 | 0.424329 |
| Enterobacteriaceae | 0 | 0.0735 | 0.008852 |
| Unclassified family within order Rhodospirillales | 0.049901 | 0 | 0.395561 |
| Helicobacteraceae | 0.084632 | 0.138091 | 0.03762 |
| Peptococcaceae | 0.204795 | 0.300682 | 0.490164 |
| Corynebacteriaceae | 0.012375 | 0.004455 | 0 |
| Clostridiaceae | 0.049103 | 0.064591 | 0.002766 |
| Erysipelatoclostridiaceae | 0.111379 | 0.133636 | 0.155458 |
| Pasteurellaceae | 0.001597 | 0.004455 | 0.002766 |
| Victivallales vadinBE97 | 0.032735 | 0.042318 | 0.066941 |
| Bifidobacteriaceae | 0.409589 | 0.135864 | 0.032087 |
| Microbacteriaceae | 0 | 0 | 0.002766 |
| Hungateiclostridiaceae | 0 | 0 | 0.014384 |
| Flavobacteriaceae | 0.000798 | 0.062364 | 0.036513 |
| Anaerofustaceae | 0 | 0.004455 | 0.008852 |
| Dermabacteraceae | 0.001597 | 0 | 0 |
| Eubacterium coprostanoligenes group | 0.009182 | 0 | 0.091283 |
| Streptococcaceae | 0.118166 | 0 | 0 |
| Staphylococcaceae | 0.002794 | 0 | 0 |
| Burkholderiaceae | 0 | 0.004455 | 0 |
| Defluviitaleaceae | 0.004391 | 0.006682 | 0 |
| **Bacterial genus** |  |  |  |
| Mucispirillum | 0.125751 | 0.198227 | 0.379517 |
| DTU089 | 0.043913 | 0 | 0 |
| Sellimonas | 0.130941 | 0.084636 | 0.025449 |
| Victivallis | 0.039123 | 0.071273 | 0.154905 |
| Oxalobacter | 0.012375 | 0.022273 | 0.088517 |
| Butyricimonas | 0.027545 | 0.095773 | 0.099582 |
| Incertae Sedis | 0.277051 | 0.066818 | 0.352962 |
| Tyzzerella | 0.021957 | 0.033409 | 0 |
| Candidatus Saccharimonas | 0.25749 | 0.211591 | 0.487397 |
| Oscillospirales UCG-010 | 0.148506 | 0.2205 | 0.494036 |
| Unclassified genus within order Oscillospirales | 0.09102 | 0.133636 | 0.188652 |
| Muribaculaceae | 0.282241 | 0.080182 | 0.044259 |
| Acinetobacter | 0.001996 | 0.017818 | 0.07358 |
| Butyricicoccaceae UCG-009 | 0.103395 | 0.256136 | 0.215207 |
| Slackia | 0.044711 | 0.035636 | 0.045365 |
| Merdibacter | 0.004791 | 0 | 0.029321 |
| Treponema | 0 | 0.093545 | 0.113413 |
| Elusimicrobium | 0.001198 | 0.098 | 0.099029 |
| DTU014 | 0 | 0.017818 | 0.038726 |
| Enorma | 0.029542 | 0.062364 | 0.011065 |
| Monoglobus | 0.03034 | 0.224954 | 0.424329 |
| Sphaerochaeta | 0 | 0 | 0.009405 |
| Fusicatenibacter | 0.137727 | 0.051227 | 0.021023 |
| Escherichia-Shigella | 0 | 0.0735 | 0.008852 |
| Marvinbryantia | 0.07585 | 0.006682 | 0 |
| Lachnospiraceae FCS020 group | 0.061877 | 0.049 | 0 |
| Eubacterium hallii group | 0.36368 | 0.262818 | 0.241209 |
| CHKCI002 | 0.035929 | 0.020045 | 0.030428 |
| ASF356 | 0.002794 | 0 | 0 |
| Unclassified genus within order Rhodospirillales | 0.049901 | 0 | 0.395561 |
| Fournierella | 0.219964 | 0.445454 | 0.076899 |
| Butyricicoccaceae UCG-008 | 0.021557 | 0 | 0.041492 |
| Unclassified genus within family Erysipelotrichaceae | 0.03553 | 0.049 | 0.059749 |
| Unclassified genus within family Lachnospiraceae | 0.405996 | 0.423181 | 0.433181 |
| Flavonifractor | 0.037126 | 0.066818 | 0.382837 |
| Barnesiella | 0.008783 | 0.124727 | 0 |
| Helicobacter | 0.084632 | 0.138091 | 0.03762 |
| Lachnospiraceae_UCG-010 | 0 | 0 | 0.045918 |
| Unclassified genus within family Peptococcaceae | 0.017166 | 0.031182 | 0.369559 |
| Turicibacter | 0.251103 | 0.467727 | 0.062515 |
| Corynebacterium | 0.012375 | 0.004455 | 0 |
| Oribacterium | 0 | 0.017818 | 0.043152 |
| Clostridium sensu stricto 1 | 0.049103 | 0.064591 | 0.002766 |
| Faecalicoccus | 0.030739 | 0.004455 | 0.006639 |
| Erysipelatoclostridiaceae UCG-004 | 0.085431 | 0.051227 | 0.080219 |
| Veillonella | 0.229545 | 0.006682 | 0.01881 |
| Candidatus Soleaferrea | 0 | 0 | 0.137202 |
| Gallibacterium | 0.001597 | 0.004455 | 0.002766 |
| Unclassified genus within family Prevotellaceae | 0.290225 | 0.093545 | 0.27551 |
| Victivallales vadinBE97 | 0.032735 | 0.042318 | 0.066941 |
| Unclassified genus within family Christensenellaceae | 0 | 0.006682 | 0.190865 |
| Colidextribacter | 0.318968 | 0.118045 | 0.382283 |
| Ruminococcus gauvreauii group | 0.317771 | 0.169273 | 0.006639 |
| Bifidobacterium | 0.377652 | 0.086864 | 0.002766 |
| Rathayibacter | 0 | 0 | 0.002766 |
| Lachnospiraceae NK4A136 group | 0.009182 | 0.033409 | 0.052004 |
| Faecalitalea | 0.071459 | 0.033409 | 0.012171 |
| Ruminiclostridium | 0 | 0 | 0.014384 |
| Lachnospiraceae UCG-008 | 0 | 0.044545 | 0.131116 |
| Unclassified genus within family Barnesiellaceae | 0.215174 | 0.414272 | 0.127797 |
| Erysipelothrix | 0 | 0.004455 | 0 |
| Unclassified genus within family Flavobacteriaceae | 0.000798 | 0.062364 | 0.036513 |
| Lachnospiraceae | 0.005589 | 0.013364 | 0.024342 |
| Clostridioides | 0 | 0.006682 | 0 |
| Anaerofustis | 0 | 0.004455 | 0.008852 |
| Clostridium innocuum group | 0 | 0.006682 | 0.022683 |
| Muribaculum | 0 | 0.008909 | 0 |
| Mailhella | 0.047107 | 0.077954 | 0.317002 |
| Brachybacterium | 0.001597 | 0 | 0 |
| Aeriscardovia | 0.031937 | 0.049 | 0.029321 |
| Erysipelatoclostridium | 0.025949 | 0.082409 | 0.023789 |
| Peptococcus | 0.187628 | 0.2695 | 0.120605 |
| Eubacterium brachy group | 0 | 0.011136 | 0.004979 |
| Anaerotruncus | 0 | 0 | 0.060302 |
| Eubacterium coprostanoligenes group | 0.009182 | 0 | 0.091283 |
| Erysipelotrichaceae UCG-003 | 0 | 0 | 0.051451 |
| Anaerosporobacter | 0.022356 | 0.044545 | 0 |
| Paludicola | 0.165273 | 0 | 0 |
| Harryflintia | 0 | 0 | 0.006639 |
| Anaerofilum | 0 | 0.035636 | 0 |
| Streptococcus | 0.118166 | 0 | 0 |
| Agathobacter | 0.034731 | 0 | 0.014937 |
| Staphylococcus | 0.002794 | 0 | 0 |
| Burkholderia-Caballeronia-Paraburkholderia | 0 | 0.004455 | 0 |
| Unclassified genus within family Desulfovibrionaceae | 0.006387 | 0 | 0.016597 |
| Anaerostipes | 0.013972 | 0 | 0 |
| Defluviitaleaceae_UCG-011 | 0.004391 | 0.006682 | 0 |
| Dielma | 0 | 0.004455 | 0 |

**
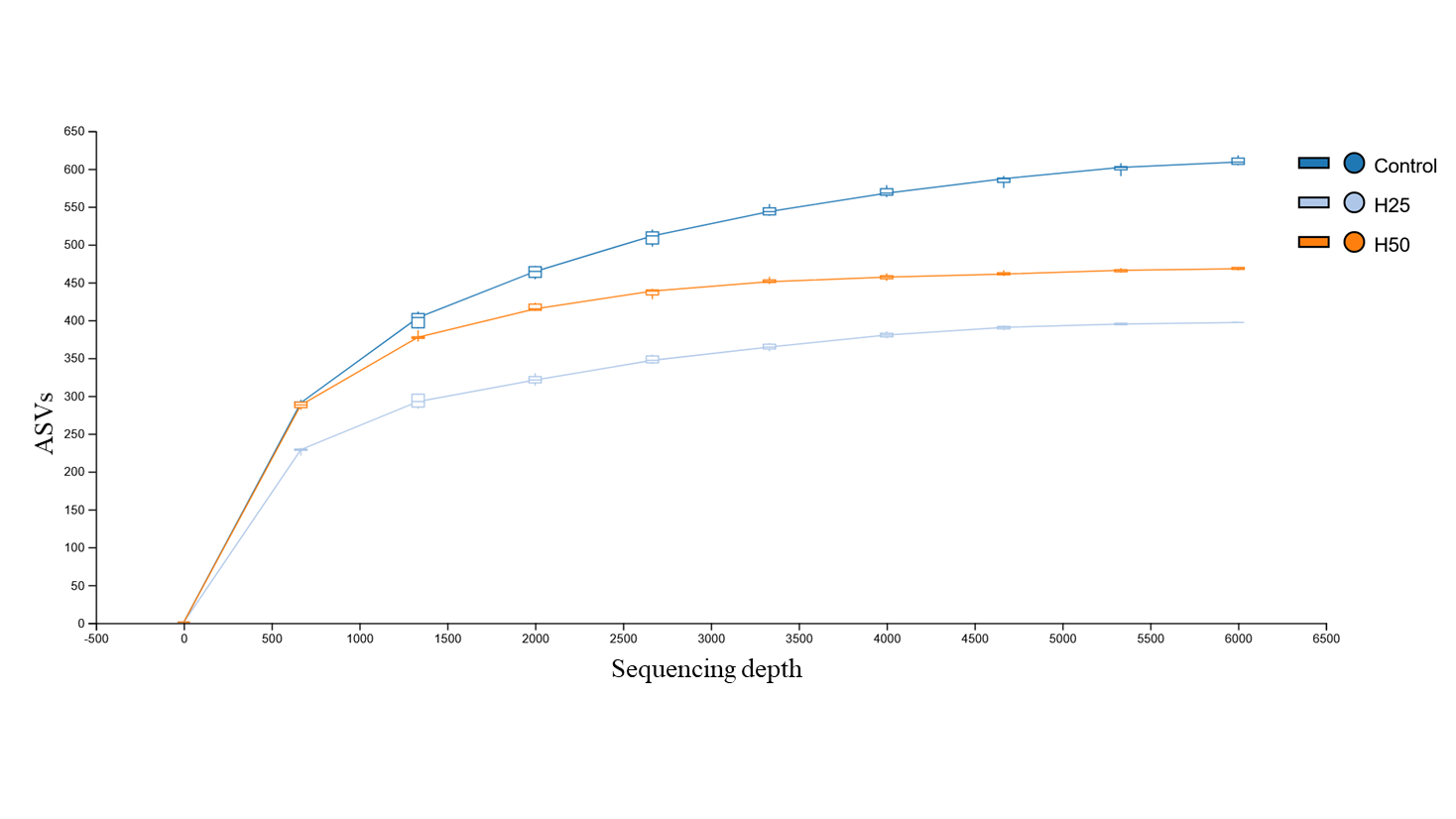
Figure S1.** Rarefaction curves representing the sequencing depth (number of reads) and the number of ASVs (sequence variants) found in the cecal content of hens.
